# The Sustainability and Scalability of Private Sector Sanitation Delivery in Urban Informal Settlement Schools: A Mixed Methods Follow Up of a Randomized Trial in Nairobi, Kenya

**DOI:** 10.3390/ijerph17155298

**Published:** 2020-07-23

**Authors:** Jedidiah S. Snyder, Graeme Prentice-Mott, Charles Boera, Alex Mwaki, Kelly T. Alexander, Matthew C. Freeman

**Affiliations:** 1 Gangarosa Department of Environmental Health, Emory University, Atlanta, GA 30322, USA; jedidiah.snyder@emory.edu (J.S.S.); graeme.prenticemott@gmail.com (G.P.-M.); 2 Consultant, Nairobi 00100, Kenya; boeracharles025@gmail.com; 3 Safe Water & AIDS Project, Kisumu 40100, Kenya; alex@swapkenya.org; 4 CARE, Atlanta, GA 30307, USA; kelly.alexander@care.org

**Keywords:** sanitation, school, informal settlements, sanitation service delivery, private sector provision

## Abstract

There are considerable challenges to achieving the Sustainable Development Goals’ target of universal access to basic sanitation in schools. Schools require safe, clean, and sex-segregated facilities for a large number of students. Robust and affordable solutions are needed to address the economic, spatial, social, institutional, and political factors which contribute to poor sanitary conditions in informal settlements. In 2015, we undertook a randomized controlled trial to assess the feasibility of private sector sanitation delivery (PSSD) in 20 primary schools, in informal settlements of Nairobi, Kenya. Our preliminary evaluation after one year of service delivery suggested that PSSD of urine-diverting dry latrines with routine waste collection and maintenance provided a feasible, lower-cost alternative to the government standard delivery (GSD) of cistern-flush toilets or ventilated improved pit latrines. We conducted a mixed-methods follow-up study to assess sanitation delivery over 3–4 years and investigate prevailing drivers and barriers that may influence the scalability of PSSD. The conditions of newly constructed and rehabilitated GSD facilities diminished quickly, reverting to the conditions of existing facilities, indicating lower sustainability compared to sanitation delivered from the private sector. Barriers in financial aspects related to the ongoing implementation of PSSD emerged, particularly among public schools, and few were able to pay for continued service. Our study demonstrates that the engagement of the private sector may lead to improvements in affordable, safely managed sanitation for schools and their students. Yet, to reach a sustained scale, additional guidance is needed on how to develop these partnerships, streamline procurement and contracting processes, and incorporate appropriate financing mechanisms.

## 1. Introduction

It is estimated that 5.3 billion people lack access to safely managed sanitation services, defined as “a system that separates human excreta from human contact at all steps of the sanitation service chain from toilet capture and containment through emptying, transport, treatment (in-situ or off-site) and final disposal or end use” [1]. Inadequate sanitation systems exist in many parts of the world, and are especially prominent in urban informal settlements [2]. Sanitation coverage may exist in established informal settlements [1], however, economic, spatial, social, institutional, and political factors contribute to poor sanitary conditions [3].

Informal settlements are typically located on the city periphery or beyond its formal boundaries, making them difficult to reach with basic service infrastructure [3]. Sanitation options are limited due to high-density living and limited land availability [4]; narrow streets which restrict access to infrastructure for maintenance or repairs [2,5]; and the poor durability of construction materials, which hinders sustainable service delivery [6]. In general, informal settlements are established on low-value, unfavorable areas and high water tables, unstable soil formation, and natural hazards such as flooding or landslides associated with these areas make traditional infrastructure systems (such as pit latrines or septic systems) inappropriate or expensive to construct [7,8,9].

The provision of safe water and hygienic sanitation facilities within schools has been shown to improve child health, decrease school absenteeism, enhance girls’ attendance and retention, and generally improve the quality of life and learning environment [10,11,12]. As such, schools are targeted for ‘universal and equitable access’ as part of the Sustainable Development Goal (SDG 6) to achieve sanitation and water for all by 2030 [13]. Robust and affordable solutions are required for providing healthy environments in school [14]. The challenges of safe sanitation systems in urban informal settlements are amplified in school settings, due to inadequate funding or the low prioritization of water, sanitation, and hygiene (WASH) programs [15,16], along with providing facilities that are sex-segregated [14,17] and adequate in both quantity and quality, to encourage student use [18]. The delivery of school-based WASH interventions alone does not guarantee positive health outcomes [19]. Implementing effective and sustainable WASH services in schools involves many factors. School sanitation facilities, in particular, are prone to disrepair and inadequate investments in operations and maintenance, and monitoring for functionality likely hinders the quality of services delivered [16,20,21].

The majority of Kenya’s urban population live in informal settlements [22], and Nairobi hosts one of the largest informal settlements in Africa [23]. In 2015, we undertook a randomized controlled trial, to assess the feasibility of private sector service delivery of sanitation in primary schools in informal settlements of Nairobi, Kenya [24]. This study was conducted in response to the limited evidence available on the effect of alternative modalities of sanitation service delivery, to overcome challenges among schools in informal settlements. The study compared private sector sanitation delivery (PSSD) of urine-diverting dry sanitation (with routine waste collection and maintenance) to government standard delivery (GSD) of cistern-flush toilets or ventilated improved pit latrines in 20 schools. Sanitation services were evaluated based on facility maintenance, toilet use, exposure to fecal contamination, and cost. Our preliminary evaluation suggested that PSSD provides a feasible, lower-cost alternative to the delivery of sewage sanitation in Nairobi informal settlements, where feasibility was defined as “the ability to provide sustained sanitation services that were similar or superior to standard government sanitation approaches” [24]. However, evaluations beyond the first year of implementation were required to better understand the sustainability of sanitation service delivery and the scalability of PSSD in the urban school environment.

We undertook a mixed-methods study to evaluate the following questions: How do the conditions of different school-based sanitation modalities (i.e., PSSD and GSD) compare over a multi-year follow up period? Moreover, what prevailing drivers and barriers may influence the scalability of PSSD in schools? The purpose of the study was to inform policy and guidelines for the provision of safely managed sanitation and clean and healthy environments in Kenyan urban informal settlement schools, and potentially elsewhere.

## 2. Materials and Methods

### 2.1. Study Design

**Figure 1:**
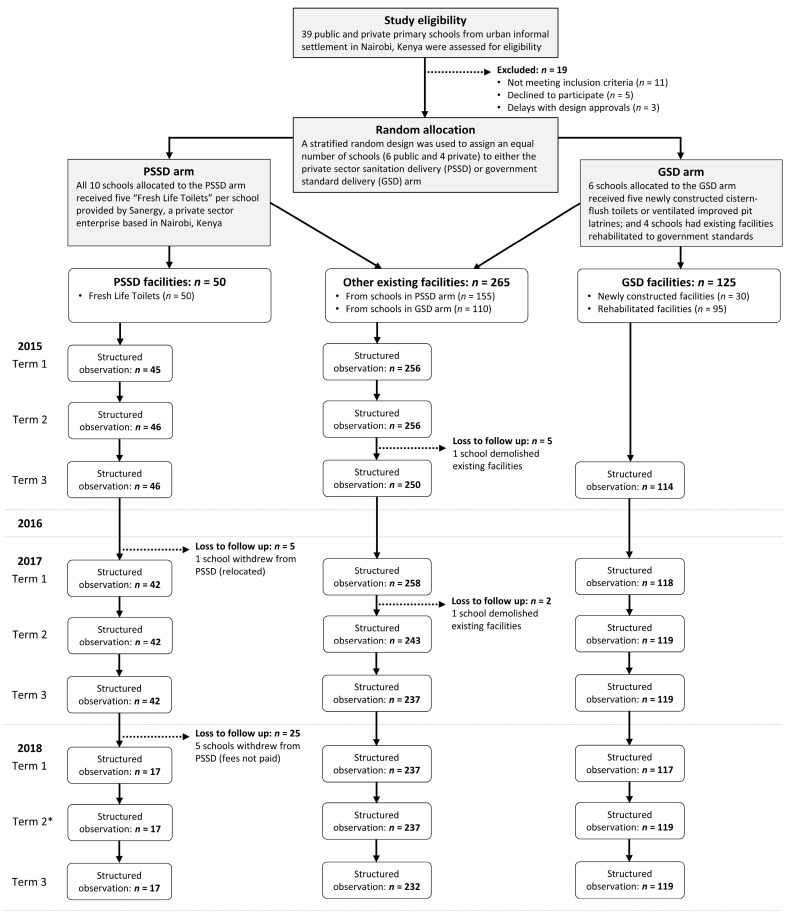
Study flow diagram. * Key informant interviews (*n* = 30) from schools in PSSD arm

This sub-study was conducted as part of a parent project—SWASH+ (School Water, Sanitation and Hygiene plus Community Impact)—which ran between 2006 and 2019, as an action-research and advocacy project focused on increasing the scale, impact, and sustainability of school WASH interventions in Kenya [25]. The study involved 20 primary schools in informal settlements of Nairobi, Kenya. Details related to the trial are published elsewhere [24]. In summary, we randomly selected and assigned 10 schools (6 public, 4 private) to receive PSSD and 10 schools (6 public, 4 private) to receive GSD (Figure 1). Schools allocated to the PSSD arm received five “Fresh Life Toilets” per school (referred to as PSSD facilities herein) provided by Sanergy, a private sector enterprise based in Nairobi, Kenya [26]. PSSD facilities were urine-diverting dry latrines, with cartridges that collected the waste. Service delivery included daily waste removal and routine maintenance visits to repair the facilities, as needed. Schools allocated to the GSD arm received sanitation packages typical of the Government of Kenya’s provision of school sanitation—cistern-flush toilets connected to the municipal septic system or ventilated improved pit latrines. Six schools allocated to the GSD arm received five newly constructed GSD facilities, and four schools allocated to the GSD arm had existing GSD facilities rehabilitated. The summarized details of PSSD and GSD can be found in Table 1.

Structured observations of sanitation facility conditions were conducted three times per year (at the end of each school term) during a 3–4 year period; beginning in January 2015 and concluding in October 2018. No data were collected during the 2016 school year. Key informants interviews (KIIs) were carried out in the second term of the final year of this study (July to August 2018), in the 10 schools enrolled within the intervention arm which received PSSD. Research was conducted as part of a collaborative approach between Emory University, Cooperative for Assistance and Relief Everywhere (CARE), Sanergy, the Kenyan Ministry of Education, and the Nairobi City Council Department of Education.

### 2.2. Structured Observations

We conducted structured observations of sanitation facility conditions during unannounced termly school visits. These visits included visual inspections of the facilities, in accordance with a prescribed checklist, to assess for usability and maintenance. Sanitation facility usability was measured using WHO/UNICEF’s Joint Monitoring Programme for Water Supply and Sanitation (JMP) WASH in schools definition, which refers to toilets/latrines that are: (1) accessible to students—doors are unlocked or a key is available at all times, (2) functional—the toilet is not broken, the toilet hole is not blocked, and water is available for flush/pour-flush toilets, and (3) private—there are closable doors that lock from the inside and no large gaps in the structure at the time of the observation [17]. For facility maintenance, we assigned values to five binary (yes/no) conditions collected during facility observations: (1) availability of cleaning materials, (2) absence of flies, (3) absence of odor, (4) absence of visible feces, and (5) absence of urine/stagnant water. These values were summed to create a maintenance score for each facility ranging from 0 (dirty) to 5 (clean). Affirmative responses were assigned one point. Each toilet/latrine block and compartment were provided unique identifiers to allow for a longitudinal analysis. Field survey data was collected electronically on password-protected mobile phones, and stored securely using Open Data Kit [27].

Sanitation facilities were stratified into three groups for a longitudinal comparison: (1) 50 Fresh Life Toilets provided to the 10 schools in the PSSD arm (PSSD facilities), (2) 125 newly constructed and rehabilitated cistern-flush toilets or ventilated improved pit latrines provided to the 10 schools in the GSD arm (GSD facilities), and (3) the 265 non-intervention facilities from the 20 trial schools (other existing facilities) (Figure 1). Sanitation facilities were lost to follow up if they were demolished or PSSD was withdrawn during the course of the follow up period. Sanitation facilities that changed designation to a non-student facility were excluded from our analysis. The proportion of usable sanitation facilities (and constituents of usability) and average maintenance scores and their 95% confidence intervals (CI) were determined for sanitation facility groups at each termly school visit. Analyses were conducted in SAS 9.4 (SAS Institute Inc., Cary, NC, USA).

### 2.3. Key Informant Interviews

KIIs were conducted using qualitative research principles [28]. Interview questions were intended to elicit narratives from the respondents regarding the scalability (e.g., key drivers and barriers in implementation) of PSSD in schools formed over the 3–4 years of the trial. Two administrative representatives were targeted for interviews for each public school (e.g., head teacher and board of management member) and private school (e.g., head teacher, and either a founder, owner, or director). In all schools, teachers selected and trained as champion teachers and school staff with sanitation facility cleaning responsibilities (e.g., caretakers) were also targeted for interviews. KIIs were conducted by two Kenyan researchers in the language most comfortable for the informant (English/Swahili) and transcribed and translated directly into English. In order to improved data quality, interviewers conducted joint interviews with a subset of participants. For quality assurance, samples were randomly selected from two interviews and transcribed into Swahili before translation into English, and both methods were compared.

Transcripts of KIIs were analyzed in MAXQDA (version 12, VERBI Software, Berlin, Germany). Inductive coding began by listening to interview recordings. Data codes were refined through the subsequent transcription of English-language interviews and dedicated data coding analysis, with coded segments stratified by school, respondent category, and specific code. The main themes were identified on the basis of occurrence and explanatory value within coded segments. Thematic data was synthesized into narrative form. To aid in this synthesis, we utilized the “SERVQUAL model”, which is a multi-dimensioned research tool traditionally applied to capture service quality and customer satisfaction [29]. Specifically, we applied the five dimensions of the SERVQUAL model (reliability, tangibles, empathy, responsiveness, and assurance), to categorize key drivers and barriers for PSSD in trial schools. An additional dimension was added to categorize financial aspects (Table 2).

### 2.4. Ethics

Ethics approval was granted by the Institutional Review Board at Emory University (IRB00079064), and the Ethical Review Committee at the Great Lakes University of Kisumu (GREC/191/01/2015). Participation in the study did not subject participants to any excess risk. Study staff ensured that representatives targeted for KIIs fully understood that responses would not in any way affect the intervention status of their school.

## 3. Results

### 3.1. Sustainability of Sanitation Delivery in Urban Primary Schools

#### 3.1.1. Survey Results

**Figure 2:**
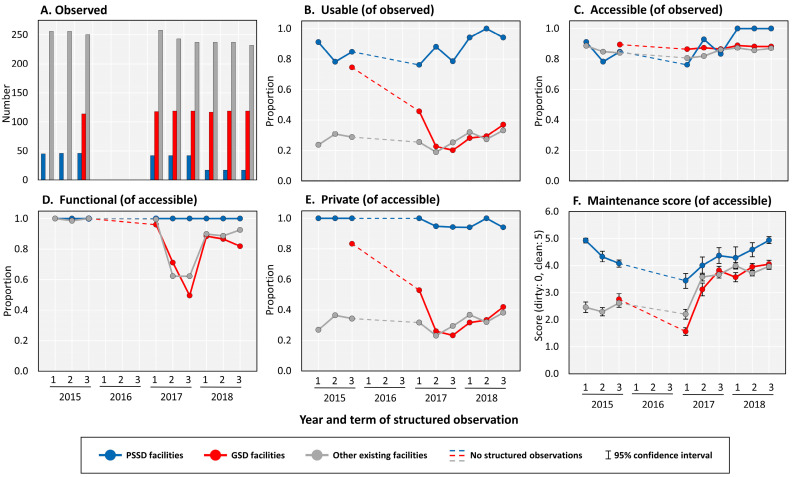
Comparisons of sanitation delivery modalities in urban informal settlement schools. (**A**) Structured observations of private sector sanitation delivery (PSSD) facilities, government standard delivery (GSD) facilities, and other existing facilities from our 20 trial schools. (**B**) Usable refers to toilets/latrines that are: (**C**) accessible to students—doors are unlocked or a key is available at all times, (**D**) functional—the toilet is not broken, the toilet hole is not blocked, and water is available for flush/pour-flush toilets, and (**E**) private—there are closable doors that lock from the inside and no large gaps in the structure at the time of the observation [17]. (**F**) Average maintenance scores, ranging from 0 (dirty) to 5 (clean), are composed of the following variables for each facility: availability of cleaning materials, absence of flies, absence of odor, absence of visible feces, and absence of urine/stagnant water.

All trial schools (*n* = 20) had three termly visits completed in the school years 2015, 2017, and 2018 (Figure 1). As previously published [24], newly constructed and rehabilitated facilities for schools in the GSD arm were not complete until late 2015. Accordingly, no structured observations were conducted for these GSD facilities until Term 3 of the same year. Over the course of the follow up period, six of the 10 schools in the intervention arm withdrew from PSSD. One private school moved to a new location and withdrew after declining to pay to relocate the facilities, and five public schools were withdrawn after failure to pay service fees—resulting in 30 of 50 (60%) PSSD facilities loss to follow up. Longitudinal trends in usability and maintenance scores of PSSD facilities, GSD facilities, and other existing facilities from our 20 trial schools are plotted in Figure 2.

#### 3.1.2. Usability

At the final visit, 44 (37%) of GSD and 77 (33%) of other existing facilities were considered usable according to the JMP definition, compared to 16 (94%) of PSSD facilities from the four schools still receiving PSSD. Across all terms of observation, the average proportion (±SD) of facilities that had doors that were unlocked or a key was available for student use (i.e., accessible) was 90% (±9.4%) for PSSD, 88% (±1.2%) for GSD, and 85% (±2.6%) for other existing facilities. All accessible PSSD facilities were functional during observations. In contrast, the functionality of GSD facilities declined within each school year (i.e., from Term 1 to Term 3)—with increases in functionality between school years (i.e., from Term 3 to Term 1 of the next year). We observed a steady decline in GSD facilities that had closable doors that lock from the inside and/or had no large gaps in the structure (i.e., private) at the time of the observation. During the first observation of GSD facilities, 85 (83%) of the facilities accessible to students were private, compared to a low of 24 (23%) just two years later. In the last two school years of observations, the average proportion (±SD) of facilities that were private was 96% (±3%) for PSSD, 35% (±11%) for GSD, and 32% (±5%) for other existing facilities.

#### 3.1.3. Maintenance Score

PSSD facilities were cleaner compared to GSD facilities across all seven terms of comparable observations. On many occasions (Term 1 and 2 of 2017 and Term 1 of 2018), GSD facilities were observed to be the dirtiest among the three facility groups. Average maintenance scores increased during the last two school years for both PSSD and GSD facilities—with slight decreases in scores between school years.

### 3.2. Exploring Key Drivers and Barriers of Private Sector Service Delivery of Sanitation in Schools

#### 3.2.1. Interview Results

Thirty KIIs were carried out in the 10 trial schools enrolled within the PSSD intervention arm. At the time of the interviews (Term 2, 2018), only four schools (1 public, 3 private) were still receiving PSSD (Figure 1). School administration was represented by one head teacher or one board of management member for each public school in the intervention arm. For private schools, school administration was represented by one head teacher and either a founder, owner, or director. Champion teachers (*n* = 7) and caretakers (*n* = 9) were also interviewed from these trial schools. Key findings of drivers and barriers for PSSD, categorized by dimensions of the service quality (reliability, tangibles, empathy, responsiveness, financial aspects, and assurance), are summarized below, and in Table 2.

#### 3.2.2. Reliability

Reliability is defined as “the ability to perform the promised service dependably and accurately” [29]. For PSSD, we considered this to involve steps of the sanitation service chain—from toilet capture and containment to emptying and waste removal from the school. Following the addition of PSSD, respondents of private schools reported improvements in the ability to provide safe sanitation to students. Two of the four private schools were located in areas without access to sewerage, and had no existing sanitation facilities within the school compound prior to PSSD. Representatives of schools with sewerage access identified sewage blockages and damage to water supply plumbing as frequent and serious issues. These blockages were reported to usually occur due to users disposing of foreign materials in the toilets. While such practices also impacted PSSD facilities, the issue was limited to a single waste collection cycle and did not damage the facility. The regular removal of waste performed by the service provider reduced the schools’ responsibility concerning safely managed waste and relieved certain associated burdens to the school. One respondent identified risks to students’ health related to pit latrine exhaustion, because residual waste would be left throughout the school compound following the procedure. Respondents who preferred PSSD commonly accompanied this preference with expressions involving environmental protection.

The mode of the toilets is the best in value, because we don’t have a sewer line, a government sewer line near us, so we cannot build another toilet…We had a toilet, it was a normal toilet, but now when you poo or you pee, they have a pipe, channeled to the river. We used to do that. We as teachers, and the leaders, and the head teacher and manager, we used to feel this is not healthy. Because we are contaminating the environment.

—Head teacher of a private school that maintained PSSD services

Some schools reported that residents from surrounding communities were permitted to use school sanitation facilities during weekends and academic breaks. In the case of PSSD, such use would overfill the storage capacity of the facilities over the course of a weekend. Subsequently, this deterred student use and, in some cases, led schools to have caretakers remove waste cartridges from the facilities. Community users also tended to fail to properly dispose of waste in terms of urine diversion and sawdust application after defecation. Some student users experienced similar challenges related to waste disposal, sometimes defecating into the urine container or clogging it with sawdust, or defecating on the latrine floor. These challenges resulted in an increased burden for caretakers and/or waste collection problems. In some cases, waste collectors would suspend collection until the school remedied the situation.

#### 3.2.3. Tangibles

Tangibles are defined as “the appearance of physical facilities, equipment, personnel and communication materials” [29]. Themes related to the maintenance and operation of PSSD facilities were considered for this dimension. The majority of caretakers identified PSSD facilities as easier to clean compared to flush toilets and pit latrines. However, barriers in maintaining the operation of PSSD facilities were reported. During the first year of the trial, schools were provided sawdust as part of the intervention. This was an essential component of PSSD facilities, reducing odor, minimizing the breeding of larvae in feces, and contributing to the processing of collected waste into manure. After the first year, school administration generally varied in their determination of purchasing sawdust.

No, the school never bought, immediately the sawdust got finished…it reached a point I came and informed the head teacher that the Fresh Life people had said that they were not to supply any more sawdust. It is the school which will be taking care of this and so you will have to buy. Actually, [the head teacher] didn’t refuse, he just said, “Oh, it’s okay,” then we parted ways, each person back to his area of work. If you get to him next time, he tells you “We will look into that.” Just that. So it went on like that until it reached a point everything had to be stopped.

—Caretaker of a public school that withdrew PSSD services

In general, school administration expressed difficulties retaining caretakers, given an inability to offer competitive wages. One administrator reported the need, in the occasional absence of a regular caretaker, to engage facility cleaning services on a per-task basis, or hiring a resident from the surrounding area and paying a one-time wage. After installation of PSSD facilities, two schools, which had previously relied on community sanitation facilities, assigned cleaning duties to students. In other schools, the installation of PSSD facilities increased the workload for caretakers, and this addition was perceived by caretakers to be supplementary to the existing scope of work and wages, with some schools increasing cleaning staff. Some caretakers were resistant to incorporate cleaning the PSSD facilities into their work priorities. In response to these challenges, several school administrators expressed a desire that the service provider also provide cleaning services of the PSSD facilities, while acknowledging that this would likely imply an increase in the current service fee.

#### 3.2.4. Empathy

Empathy is defined as “the provision of caring, individualized attention to customer” [29]. Services to address the accessibility for individuals (e.g., girls, disabled and small children) and training for participation indicated empathy. Respondents across all schools indicated satisfaction with PSSD, due to the rapid increase in the number of school sanitation facilities. The addition of PSSD alleviated the problem of student queuing at sanitation facilities, noted by respondents as a persistent challenge. Several administrators reported that the addition of PSSD enabled the sex-segregation of sanitation facilities to be instated when previous facilities had not been sex-segregated. In addition, respondents indicated a preference for assigning PSSD facilities to upper classes of girls: they were perceived to be more responsible users, would benefit more from the apparent improved hygienic design, and were thought to provide better accommodation and privacy to girls experiencing menses. In schools without existing sanitation facilities, students were required to use the nearest available facilities outside of the school compound (e.g., community sanitation facilities). School representatives identified risks to the well-being of students when it is necessary for them to frequently leave the school compound. These dangers included traffic-related injury when schools are located nearby major roadways (which was reported to have occurred) and the increased vulnerability of female pupils to sexual violence.

Before, children could go outside, but now they are inside. It is even eas [ier] to monitor the children…because you know there are people who even go and don’t come back, even at break time. But because they are here, it is safe. They are safe.

—Teacher of a private school that maintained PSSD services

Respondents reported the positive evaluations of the training of champion teachers instated by the service provider. However, school representatives emphasized the importance of training newly enrolled students that may be unaware of proper dry sanitation facility use. Overall, challenges arose concerning the use of the PSSD facilities by younger children. School representatives theorized that small children experienced these challenges due to the design of the squat plate and the size of the waste container openings. For this reason, schools preferred not to assign younger classes to these facilities.

#### 3.2.5. Responsiveness

Responsiveness refers to “the willingness to help customers and to provide prompt service” [29]. For PSSD, the ability to accommodate services for the school setting was considered as responsiveness. PSSD in schools presented unique servicing needs—facilities in schools experienced high-volume use at select time points throughout the day. Students often arrived early to use the facilities before classes began, or used them throughout the day during class breaks. For this reason, school respondents placed high value on regular waste collection. In addition, these respondents reported satisfaction when the service provider was responsive to requests for accommodating school class schedules, by collecting waste at specific times of the day, along with requests for maintenance repairs or unscheduled waste collection if PSSD facilities became full.

And about collection of wastes, it is good they never delay or miss to come though. There were two occasions they delayed but when I called them, immediately they sent their people to come and empty and so I feel that they are so much concerned.

—Board of management member of a private school that maintained PSSD services

High value was placed on communication hierarchies, especially in public schools. School staff members responsible for operating PSSD facilities (e.g., champion teachers and caretakers) interfaced most frequently with service provider employees. However, administrators expressed displeasure when this interface was used as a communication channel for important information regarding waste collection. For example, if feces or urine were improperly deposited due to incorrect use by students, waste collectors would decline to carry out waste collection on some occasions. In such instances, school administrators reported the need for direct communication with the service provider.

#### 3.2.6. Financial Aspects

Financial aspects refer to available funds, where they come from, and how they are spent. Representatives of private schools expressed a willingness and ability to secure funding needed to cover PSSD service fees. Financing methods in private schools included student school fees, sponsorship from a non-governmental organization, fundraising efforts with parents, and even the collection of fees from community members using PSSD facilities. All public schools reported the government-sponsored, Free Primary Education program to be the exclusive funding source for school sanitation. Respondents at two public schools mentioned the operation of a pre-school as an additional financing method. However, county government assumed control of these pre-schools, which affected the school’s ability to pay service fees. Of six public schools, only three made payment of PSSD service fees for at least one term, while the remaining made no payments at any point. Service continued for non-paying schools for much of 2017—during a period of protracted negotiation over the service fee amount. Eventually, the service was discontinued for all but one public school, which continued to make payments in full. Administration from this school reported that it was able to pay for service fees by renting out its facilities. Other public schools, when asked about this financing method, indicated that it was infeasible, due to the complexity of a process involving city or county government, where revenue may not necessarily go to the school, and due to concerns of damage to school facilities. Each of the withdrawn public schools reported unwillingness or inability to pay service fees as the first and, in some cases, only reason, for withdrawing from PSSD.

And you see when this money from the government comes, it comes with its—with the items. You spend that money per item. If it’s for electricity, if for water, if for sanitation, you cannot overspend…and so if you were to squeeze Fresh Life payments in this—You see for sanitation, it’s not about the toilets alone. We have to buy the sanitary towels for the kids. We have to buy the soap, cleaning equipment for the toilets, you see, so all those things are supposed to be bought.

—Board of management member of a public school that withdrew PSSD services

Prior to the implementation of PSSD, two private schools had been paying fees for students to use community facilities. PSSD service fees were considered less costly than this arrangement, and more suitable. All public schools had existing sanitation facilities within its own compound prior to the implementation of PSSD—as required by government policy. Accordingly, many public school administrators reported feeling no reliance on PSSD facilities, and declined to use school resources in order to pay for them. This was true even in cases where administrators explicitly indicated that the operation and maintenance of PSSD was less expensive than the school’s existing facilities.

#### 3.2.7. Assurance

Assurance is defined as “the knowledge and courtesy of employees and their ability to convey trust and confidence” [29]. Themes surrounding the procurement and contracting processes and resulting service delivery partnerships were considered for this dimension. In private schools, procurement and contracting processes involved a unilateral process, in that decision-making was contained within a single administrative entity, a school owner, director, or head office. This dynamic enabled private schools to establish and maintain partnerships with outside institutions. This is in contrast to public schools, which have several internal oversight bodies with interconnected responsibilities. In order to establish partnerships with the service provider, public school head teachers were required to seek approval, from the boards of management, school committees, and parent organizations. Head teachers expressed difficulty navigating this and, for some in this position, response to this challenge involved expressing a preference that partnerships with potential service providers be established and monitored by the county education department.

[The government] can sign that agreement. When the toilets get to the school, it’s the work of the head teacher to take care of them. Let me be supervised…even me, I fear I can’t take any help [from] any NGO. Like today, I saw one—I refused. I said no. No, no, no, no. Go back to the director [of county education], and inform the director you want to do this in schools…But I won’t take it, because I have that fear. You have to take that agreement, go with it to the board of management, read for them, ask them, before you take a step. No matter how good that NGO is. Or the agreement looks as if it help the children. I don’t want [it].

—Head teacher from a public school that withdrew PSSD services

Administrators from several schools, both public and private, reported having incomplete knowledge about the terms within the service agreement prior to signing, and based their understanding on interpersonal interactions with representatives of the service provider. At the time of enrollment into the trial, schools in the PSSD arm were informed that PSSD required no payment from the school for the duration of the first year. Prior to installation, the service provider set expectations with all schools on how much they were to pay. Despite being informed of the PSSD model, which is sustained through service fees, administrators originally perceived service in their schools as a donation. Frustration was expressed, both in public and private schools, that insufficient emphasis was placed on the requirements for service fees in subsequent years, however, during interviews, public school administrators tended to revisit this topic more frequently and give it greater importance than their counterparts in private schools.

## 4. Discussion

This was a follow-up study of our previous evaluation of the feasibility of sanitation delivered by a private sector enterprise (i.e., urine-diverting dry latrines with routine waste collection and maintenance), as compared to sanitation packages typical of the Government of Kenya’s provision of school sanitation (i.e., cistern-flush toilets or ventilated improved pit latrines where feasible) [24]. We conducted a longitudinal assessment of the conditions of different sanitation modalities delivered within primary schools in informal settlements of Nairobi, Kenya, and investigated prevailing drivers and barriers that may influence the scalability of private sector service delivery of sanitation in schools. In our 3–4 year follow up, conditions of newly constructed and rehabilitated cistern-flush toilets and pit latrines diminished quickly, reverting to the conditions of existing facilities, indicating lower sustainability compared to sanitation services from the private sector. Initial capital investments for PSSD were considerably lower than that required for rehabilitating government standard flush-type toilets and constructing new facilities (Table 1) [24]. However, barriers in financial aspects related to the ongoing implementation of PSSD emerged, particularly among public schools, and few were able to pay for the continued service.

The sustainability of gains from WASH in schools interventions are often endangered by a lack of supporting systems [20,30]. Over two years, we found the decreased usability of GSD facilities compared to PSSD facilities. The trends we observed have important policy implications because, in addition to adequate access, the functionality of WASH infrastructure over time is recognized as the most important constraint to operational sustainability [31]. The functionality of GSD and other existing facilities varied throughout the year (something not observed with PSSD), and repairs to address decreased functionality were not made until the end of the school year. The privacy of newly constructed and rehabilitated facilities worsened, with little to no evidence of repairs to restore privacy conditions. Adequate sanitary conditions in schools is important for equitable access to education [32,33]. The attendance and participation of girls, in particular, has been shown to suffer when sanitation facilities do not offer a safe, clean, and healthy environment for menstrual hygiene management [34,35,36,37]. GSD schools were unable to maintain adequate sanitation conditions, such as closable doors that lock from the inside and facilities with no large gaps in the structure. Challenges in sustaining WASH conditions that support menstruating girls have been documented, even with the provision of interventions such as menstrual hygiene management manuals covering topics including the importance of sanitation facility privacy [38]. Our findings suggest that private sector service delivery of sanitation provides better accommodation and privacy to girls during menses. However, as reported elsewhere [39,40], the accommodation for religious practices, such as ablution, should be considered when implementing dry sanitation services.

Keeping sanitation facilities clean and free of excretion is critical for reducing health risks and encouraging use [41], and in school settings, unclean sanitation facilities are likely associated with increased exposure to fecal material and pathogens to children [42,43]. Cleaner sanitation facilities have also been shown to be associated with increased use among school children [18,44,45]. Our data suggest that PSSD facilities may provide more sustained safe sanitation services compared to GSD facilities. We observed PSSD facilities to be cleaner on a consistent basis, and environmental swabs from facility surfaces during the first year of the trial were associated with lower concentrations of fecal indicator bacteria, as compared to GSD facilities [24]. Interestingly, after the first year, data suggest improved trends in the cleanliness (i.e., maintenance score) for all types of facilities. Our routine visits to evaluate sanitation conditions (three times per year) may have raised the awareness of WASH operation and maintenance activities at the school. These indirect means of enforcement, such as surveillance, have been shown to be more effective compared to other direct methods [46]. Findings from our study may further support the need of routine monitoring for the provision of sustainable WASH programs in schools [20,47].

Private sector service delivery of sanitation is a feasible compliment to meet the demand for safe sanitation in schools [24], and the engagement of the private sector could fill critical gaps in sanitation provision towards universal access [13]. Kenyan policies aim “to foster strong private sector participation and investment in creating sanitation demand and increasing uptake of appropriate products and services” [48]. We identified several preferential features of PSSD; however, the withdrawal of most public schools from the service model indicates a need to provide support and guidance to school-level decision makers, for whom such services may be suitable. A recent situation analysis of the urban sanitation sector in Kenya concluded that “the legal framework for sanitation remains fragmented and focuses on sewerage services” [49]. To achieve sustained scale, policies intended to promote and guide private sector service delivery of sanitation in schools will need to address the limited ability of public schools to establish partnerships outside of the traditional sanitation framework.

Within Kenyan standards and guidelines for school WASH infrastructure, the private sector is identified as a stakeholder providing specialized WASH operation and maintenance services, and one involved in supporting school WASH infrastructure development [50]. There is general consensus for the need for the private sector’s engagement in the WASH sector, but many policies provide little emphasis on actionable ways forward [14,51]. As indicated by our study, successful partnerships of the private sector with public schools may require education ministries to provide additional support to school-level decision makers. The ongoing sensitization of different sanitation modalities and attempts to address key barriers in their implementation are needed, to enable engagements between schools and the private sector.

### Limitations

There were several limitations to this study. First, the loss to follow up of a majority of PSSD facilities, as a result of service withdrawal, reduced our sample size throughout the course of the trial. Several schools also assigned PSSD and GSD facilities for non-student use (e.g., designated for teachers), and were not included in our observations. Second, we had hoped to explore the life cycle costs associated with the ability of schools to continue to pay for private sector services and sustained maintenance [24]. WASH-related expenditures were collected throughout the trial, in accordance to methods used in another SWASH+ sub-study [16]. However, as noted, three public schools in the PSSD arm made no payments of services fees at any point during the trial. The interpretation of reported costs would not be an accurate reflection of financial outcomes associated with PSSD, and are therefore not reported here. Third, the trial only evaluated a single private sector provider of sanitation services. Novel service-based sanitation approaches have also been piloted in refugee camp settings in Kenya [40]. These types of alternative solutions for safe and sustainable sanitation are likely applicable to other underserved populations, and should be considered for PSSD in informal settlement schools.

## 5. Conclusions

Our study demonstrates that the engagement of the private sector may lead to improvements in the affordable, safely managed sanitation for schools and their students. However, additional guidance is needed on how to develop these partnerships, streamline procurement and contracting processes, and incorporate appropriate financing mechanisms. The delivery of sanitation services through the public sector may not serve all schools, and a mix of different sanitation modalities is likely needed to achieve safe sanitation in schools. While this study adds to the case for scaling up engagement of the private sector in support of universal access to WASH, additional research is needed on the best modalities for delivery and the relevant policies that would enable scale-up.

## Figures and Tables

**Table 1:** Summary details of private sector sanitation delivery and government standard delivery of sanitation for trial schools.

Component	Private Sector Sanitation Delivery (PSSD)	Government Standard Delivery (GSD)
Delivery model	Sanitation delivery by Sanergy ^a^, a private sector enterprise based in Nairobi, Kenya	Sanitation packages typical of the Government of Kenya’s provision of school sanitation
Facility type	Prefabricated urine-diverting dry latrines, with cartridges to collect waste	Cistern-flush toilets connected to the municipal septic system or ventilated improved pit latrines ^b^
Waste removal	Waste collection team removes and replaces cartridges to dispose of waste for off-site treatment on a daily basis	Connection to sewerage and water (cistern-flush only)
Hygiene promotion	Hygiene curriculum to promote behavior change	No hygiene promotion outside of existing curriculum
Training	Training of “champion teachers” to encourage hygiene practices and proper use of facilities	None (school responsible)
Consumables	Waste cover material (sawdust)	Water (cistern-flush only)
Cleaning	None (school responsible)	None (school responsible)
Maintenance repairs	Routine maintenance visits to repair facilities, as needed	None (school responsible)
Initial cost ^c^	USD 2053 (KES 210,000) per school for five facilities and one year service delivery [24]	USD 11,489 (KES 1,169,668) per school for five newly constructed facilities [24] USD 9306 (KES 922,638) per school for rehabilitated facilities [24]
Recurring service fees	USD 292 (KES 30,000) per school for five facilities and one year service delivery ^d^	None (school responsible)

^a^ Sanergy adapted their business model (a private franchise of prefabricated toilets operated by micro-entrepreneurs throughout Nairobi’s informal settlements) in order to provide facilities for schools in the informal settlements. ^b^ One school received five ventilated improved pit latrines rather than cistern-flush toilets, due to lack of space to build a septic tank. ^c^ Waste cover material for PSSD and the initial costs for PSSD and GSD were provided by the SWASH+ project for the first year of the trial. ^d^ Service fee initially set at KES 6000 per year per facility. At the time of enrollment, funds available to public schools for “Environment and Sanitation” from the government-sponsored, Free Primary Education program were KES 50 per student [16]—or KES 46,500 per year based, on an average student enrollment of 930, for public schools allocated to the PSSD arm.

**Table 2:** Summary of key drivers and barriers for the implementation of private sector delivery of sanitation in trial schools.

Dimension of Service Quality	Attributes of Sanitation Delivery	Drivers	Barriers
^a^ Reliability: The ability to perform the promised service dependably and accurately	Safely managed waste capture, containment, and emptying	Alternative for schools located in areas without access to sewerage Resilient to sewage blockages associated with flush toilets Shorter-term waste containment compared to pit latrines of which emptying is identified as a risk to students’ health Regular waste removal reduces burden on the school Viewed favorably in the context of environmental protection	Waste containment capacity may deter student use until waste is removed Improper use of facilities can cause challenges in waste removal Waste removal may stop if terms within the service agreement are not met
^a^ Tangibles: The appearance of physical facilities, equipment, personnel and communication materials	Maintenance and operation	Caretakers perceived facilities as easier to maintain compared to traditional facilities (e.g., flush toilets and pit latrines)	School administration varied in their determination of purchasing consumables required for effective dry sanitation (e.g., sawdust) Caretakers may perceive the maintenance of non-traditional facilities (e.g., urine-diverting dry latrines) to be supplementary to existing scope of work and wages Low wages provided to caretakers may promote turnover of those trained on maintaining non-traditional facilities
^a^ Empathy: The provision of caring, individualized attention to customer	Accessibility for individuals and training	Viewed favorably in rapidly alleviating student queuing and provide sex-segregated facilities Viewed to provide better accommodation and privacy to girls during menses Alternative for schools using community facilities which may pose danger to student well-being when leaving school grounds Satisfaction associated with the training of trainer model (e.g., “champion teachers”) instated by the service provider	Newly enrolled students may be unaware of proper use of non-traditional facilities Designs were generally not conducive to proper use by younger school children
^a^ Responsiveness: The willingness to help customers and to provide prompt service	Accommodation for the school setting	Satisfaction associated with the service provider’s accommodation of school class schedules for waste collection and maintenance repairs	Communication hierarchies at schools may hinder responsiveness of the service provider
Financial aspects: Available funds, where it will come from, and how it will be spent	Service fees	School administration perceived private sector delivery of sanitation to be less expensive than traditional facilities Private schools expressed willingness and ability to secure funding for service fees Private schools preferred service fees over paying for student use of community facilities	Public schools perceived service fees as extra and prioritized existing facilities for financial resources (regardless of satisfaction) Public schools were restricted in funding and financing mechanisms to pay for service fees
^a^ Assurance: The knowledge and courtesy of employees and their ability to convey trust and confidence	Procurement and contracting processes and partnerships	Partnerships facilitated in private schools by unilateral decision-making structures	Partnerships hindered in public schools by multiple oversight bodies and administrative turnover Service delivery was originally viewed as a donation and hindered future service agreements Over reliance on interpersonal interactions hindered knowledge of service agreements

^a^ Dimensions from “SERVQUAL: A multiple-item scale for measuring consumer perceptions of service quality” [29].
